# Herbal Compatibility of Ginseng and Rhubarb Exerts Synergistic Neuroprotection in Cerebral Ischemia/Reperfusion Injury of Rats

**DOI:** 10.3389/fphys.2019.01174

**Published:** 2019-09-13

**Authors:** Wen-Ting Yang, Yong Wang, Yi-Hua Shi, Huan Fu, Zhen Xu, Qing-Qing Xu, Guo-Qing Zheng

**Affiliations:** Department of Neurology, The Second Affiliated Hospital and Yuying Children’s Hospital of Wenzhou Medical University, Wenzhou, China

**Keywords:** Ginseng, Rhubarb, cerebral ischemia/reperfusion, connexin-43, aquaporin-4, neuroprotection

## Abstract

**Objective:**

Ischemic stroke is a complex multifactorial disease caused by interactions among polygenetic, environmental, and lifestyle factors with limited effective treatments. Multi-herbal formulae have long been used for stroke through herbal compatibility in traditional Chinese medicine (TCM); however, there is still a lack of evidence due to their unimaginable complexity. Herbal pairs represent the simplest and basic features of multi-herbal formulae, which are of great significance in clarifying herbal compatibility. Here, we aim to investigate the neuroprotective effects of the herbal compatibility of Ginseng and Rhubarb on a cerebral ischemia/reperfusion (I/R) injury model of rats.

**Methods:**

Male adult SD rats were randomly divided into a sham group, a normal saline (NS) group, a Ginseng group, a Rhubarb group, and a Ginseng + Rhubarb (GR) group, a Carbenoxolone [CBX, gap junction (GJ) specific inhibitor] group, and a GR + CBX group. Each group was further assigned into four subgroups according to ischemic time (6 h, 1 day, 3 days, and 7 days). The cerebral I/R injury model was established according to the modified Zea Longa method. The Neurological Deficiency Score (NDS) was assessed by the Zea-Longa scale; the cerebral infarction area was detected by TTC (2,3,5-triphenyltetrazolium chloride) staining; and the expression of connexin-43 (Cx43) and aquaporin-4 (AQP4) were detected based on an immunofluorescence technique and quantitative real-time-PCR.

**Results:**

Compared to the I/R group, both the independent and combined use of Ginseng and Rhubarb can significantly improve NDS (*P* < 0.05), decrease the percentage of the cerebral infarction area around the infarction penumbra (*P* < 0.05) and down-regulate the expression of Cx43 and AQP4 after I/R injury (*P* < 0.05). The GR had more significant effects than that of Ginseng and Rhubarb (*P* < 0.05). Compared with the GR group, the GR + CBX group significantly improved in NDS (*P* < 0.05), and decreased the percentage of the cerebral infarction area (*P* < 0.05) and expression of Cx43 and AQP4 protein (*P* < 0.05).

**Conclusion:**

The herbal compatibility of Ginseng and Rhubarb synergistically exerts neuroprotective function during acute cerebral I/R injury, mainly through reducing the expression of Cx43 and AQP4.

## Introduction

Stroke is an acute cerebrovascular disease caused by local cerebral blood circulation disorder, which is the second cause of global human deaths (GBD 2016 Causes of Death Collaborators, 2017). According to the report published by the World Health Organization in 2017, about 6.24 million people die of stroke every year (World Health Organization, 2017). Stroke has a more serious impact in China (Gao et al., 2018). There are 1596.0 per 100,000 people suffering from stroke in China, and the incidence and mortality of stroke are 246.8/100,000 and 114.8/100,000, respectively, which causes a heavy medical and social burden (Wang et al., 2017). The incidence of stroke in China is increasing at an annual rate of 8.7% and the age of onset is becoming younger and younger (Wang et al., 2019). Stroke includes ischemic and hemorrhagic stroke, and the former is the most common subtype, accounting for 87% of all cerebrovascular accidents (Benjamin et al., 2017). Currently, intravenous alteplase and/or mechanical thrombectomy, recommended by the 2018 American Heart Association/American Stroke Association (AHA/ASA) guidelines, are effective treatments for patients with acute ischemic stroke (Powers et al., 2018). However, thrombolysis has a narrow time window and associates with lethal complications such as an intracerebral hemorrhage (Shobha et al., 2011; Wołoszyńska and Stȩpień, 2017). Mechanical thrombectomy requires rapid cerebral angiography in experienced stroke centers and qualified neurointerventional doctors. These factors largely limit their clinical use. Thus, it is necessary to find alternative therapeutics for patients with acute ischemic stroke.

Traditional Chinese medicine (TCM) has been applied in the treatment of stroke for thousands of years (Wang et al., 2011). Herbal formulas are the most common approach of TCM intervention, which is usually formed with more than two herbs to produce synergistic effects and/or reduce potentially adverse reactions (Cheng et al., 2017). Herbal pairs are the most fundamental and simplest unit of complex herbal formulae without altering their basic therapeutic characteristics (Deng et al., 2008), which represent the cornerstone of herbal compatibility (Wang et al., 2012; Jin et al., 2016). Ginseng and Rhubarb are two frequently prescribed herbs in TCM intervention for acute stroke, guided by TCM principles. Ginseng is the root and rhizome of *Panax ginseng* C. A. Meyer, which has been used as a representative tonic remedy in China and elsewhere for over 2000 years (Nah et al., 2007), and remains one of the most commonly used healing herbs for stroke (Zheng et al., 2011). The main pharmacologically active ingredients of Ginseng are Ginsenosides, responsible for most of the activities of Ginseng (Lü et al., 2009). Rhubarb is listed as the dry root and rhizome of *Rheum officinale* Baill., *Rheum palmatum* L., and *Rheum tanguticum* Maxim in the current Chinese Pharmacopeia. Extensive phytochemical research on Rhubarb has isolated and identified about 200 chemical compounds (Wang et al., 2013), such as anthraquinones, dianthrones, stilbenes, anthocyanins, flavonoids, tannins, organic acids, and chromones (Huang et al., 2007). Ginseng functions to strengthen vital Qi for brain protection aimed at its root causes, while the latter has Tongfu functions (Lu et al., 2014) for pathogenic factors aimed at manifestation. This herbal pairing thus serves to treat both the manifestation and root cause of acute stroke, according to TCM principles of treatment. Based on modern pharmacological studies, Ginseng and its active ingredients are potential neuroprotective agents in the treatment of stroke (Rastogi et al., 2015), which have multi-leveled, multi-channeled, and multi-targeted protective effects (Kim et al., 2013; Ong et al., 2015; Rokot et al., 2016). In modern pharmacological research, Ginseng and total ginsenosides could improve neurological function, reduce the volume of cerebral infarction, promote angiogenesis, and nerve regeneration in cerebral ischemia/reperfusion (I/R) injury rats (Zheng et al., 2011). *In vitro* experiments indicated that ginsenoside had anti-inflammatory, anti-oxidative stress and anti-apoptotic effects, promoting cell survival (Zhang et al., 2016; Dong et al., 2017). Our previous study showed that ginsenoside Rg1 could improve neurological injury and alleviate blood brain barrier disruption in cerebral I/R rats, and the mechanism may be related to the down-regulation of aquaporin 4 (AQP4) expression (Zhou et al., 2014). Rhubarb compound prescription can promote cerebral vascular recanalization, improve brain tissue injury, and alleviate brain cell damage caused by cerebral ischemia (Yan and Fu, 2011). Active compounds of Rhubarb root and rhizome can alleviate focal cerebral ischemia injury and have neuroprotective effects in rats (Liu et al., 2015). Our previous study indicated that Sanhua Decoction, with Rhubarb as its main component, has a significant protective effect on the neurovascular unit (NVU) in cerebral I/R injury rats, and the mechanism is mainly related to the regulation of AQP4 expression (Lu et al., 2015). However, there is lack of studies on the mechanisms of herbal compatibility in Ginseng and Rhubarb.

Brain edema is one of the most common complications of ischemic stroke, which can cause neurological deterioration and even death. Preventing and treating brain edema effectively is a key measure in reducing the mortality and disability rate (Kimberly et al., 2018). AQP4 is the most abundant and important aquaporin expressed in the nervous system (Papadopoulos and Verkman, 2013; Maugeri et al., 2016). Brain edema is closely related to the increased expression of AQP4 at the early stage of ischemic cerebral infarction. The result of studies on cerebral I/R mice with AQP4 gene knockout showed that the deletion of AQP4 gene could alleviate cytotoxic edema (Yao et al., 2015), and improve the long-term prognosis (Hirt et al., 2017) and the survival rate (Akdemir et al., 2014). Gap junction (GJ) channels span two plasma membranes to allow cells to exchange messages and material (Hervé and Derangeon, 2013). Connexin (Cx) is a family of membrane proteins that constitute the basic structure of GJ between cells. Up to now, more than 20 types of Cx have been found in mammals (Nielsen et al., 2012). Cx43 is the most common connexin in the nervous system. When it comes to ischemic necrosis, the expression of Cx43 in reactive astrocytes increases and the half-channels in the neurons open (Thompson et al., 2006; Le et al., 2014). Apoptotic information is then transmitted causing “bystander death” of adjacent cells. At the same time, adenine nucleoside triphosphate, calcium ion, cyclic adenosine phosphate, and inositol triphosphate can exchange between cells through half-channels causing further neuronal damage (Orellana et al., 2011; Gilleron et al., 2018). Inhibiting the expression of Cx43 and the opening of half-channels can therefore play a neuroprotective role. Cx43 and AQP4 are interrelated in water balance and GJ communication. The high expression of Cx43 would be decreased with the silencing of the AQP4 gene (Kong et al., 2008). In addition, the absence of Cx43 may also lead to partial loss of AQP4 (Ezan et al., 2012; Li et al., 2015). In the present study, we aim to investigate the synergistic effects of the herbal pairing of Ginseng and Rhubarb on Cx43 and AQP4 expression in cerebral I/R injured rats.

## Materials and Methods

### Ethics Statement

All experimental animals were obtained from the Shanghai Laboratory Animal Center (License number: SCXK (Hu), 2010-0002). The animal experiment protocol was approved by the local ethics committee of the Wenzhou Medical University (Approval number: wydw2015-0148) and was performed in strict accordance with its guidelines. Anesthesia was used to sacrifice the animals at the end of the experiment. The utmost efforts were made to reduce the number of experimental animals and to minimize animal suffering.

### Animals and Groups

Adult male Sprague-Dawley (SD) rats weighing between 250 and 280 g were housed at 23 ± 2°C in relative humidity of 50 ± 10% with a 12 h light/dark cycle, and with free access to food and water.

Healthy SD rats were randomly divided into seven groups: sham group, normal saline (NS) group, Ginseng group, Rhubarb group, Ginseng + Rhubarb (GR) group, Carbenoxolone (CBX, GJ specific inhibitor) group, and GR + CBX group. The sham group, NS group, Ginseng group, Rhubarb group, and GR group were further assigned into four subgroups according to 6 h, 1 day, 3 days, and 7 days the time points after I/R injury, respectively. There was only one subgroup (1 day after I/R) in CBX group and GR + CBX group. There were 15 rats in the subgroup of each time point.

### Drug Administration

Ginseng (Guangdong Yifang Pharmaceutical Co., Ltd., approval number: country medicine accurate character 7032361) and Rhubarb (Guangdong Yifang Pharmaceutical Co., Ltd., approval number: country medicine accurate character 6112271) were granules and were dissolved in distilled water at 100°C. Carbenoxolone (Abcam (Shanghai) Trading Co., Ltd.) was dissolved in NS at a concentration of 10 μg/μl. After conversing human doses to rat equivalent doses based on body surface area (Food and Drug Administration, 2005; Chen, 2011; State Pharmacopoeia Commission, 2015), a Ginseng dose of 0.7 g/kg was administered intragastrically to the rats in the Ginseng group. A Rhubarb dose of 0.2 g/kg was administered intragastrically to the rats in the Rhubarb group. Ginseng and Rhubarb (2:1) doses of 0.7 g/kg:0.2 g/kg were administered intragastrically to rats in the GR and GR + CBX group. The same of NS volume was administered intragastrically to rats in the sham group, NS group, and the CBX group instead. Administration of Ginseng, Rhubarb, GR, or NS was performed once a day starting at 3 days before the operation until the rats were sacrificed. Intracerebroventricular injection of CBX at a dose of 25 μg/kg was performed on rats in the GR + CBX group and CBX group at 0.5 h before the operation (Zhang et al., 2013; Wang et al., 2015).

### Ischemia/Reperfusion Model

Rats were anesthetized with 4% chloral hydrate (3 ml/kg) intraperitoneally and then received an operation according to the modified Zea Longa method (Longa et al., 1989). In brief, the left common carotid artery (CCA), internal carotid artery (ICA), and external carotid artery (ECA) of the rats were isolated *via* the midline incision of the neck. A 0.26 mm diameter monofilament nylon suture with a rounded tip (Beijing Cinontech Co., Ltd., Beijing, China) was introduced into ECA lumen and then gently advanced into the ICA in order to block the origin of the middle cerebral artery (MCA). For the rats in the sham group, the nylon suture was inserted into ECA lumen but not advanced into the ICA. After 2 h of occlusion, the nylon suture was withdrawn to establish reperfusion. After arousal from anesthesia, the rats were returned to cages with free food and water.

### Neurological Deficiency Score

Rats were examined for a neurological deficiency score (NDS) at 6 h, 1 day, 3 days, and 7 days after I/R using a five-tiered grading system according to Longa et al. (1989) as follows: 0, no deficit; 1, failure to extend contralateral forepaw; 2, spin longitudinally; 3, falling to the contralateral side; 4, unable to walk spontaneously. Rats with a score between 1 and 3 were selected for the study.

### Triphenyltetrazolium Chloride Staining and Infarction Area Assessments

Rats were anesthetized with 4% chloral hydrate (3 ml/kg) intraperitoneally at 6 h, 1 day, 3 days, and 7 days after I/R, and the brains were removed after profusion with NS. A brain slicer was used to coronally section the brains at 2 mm intervals from the frontal pole. The slices were incubated with 1% triphenyltetrazolium chloride (TTC) solution for 15 min at 37°C in the dark, and then immersed with 4% paraformaldehyde. The infarct areas and total area on each slice were calculated using Image-Pro Plus 6.0 software, and then expressed as the percentage of infarction in the total area.

### Immunofluorescence Staining

The rats were anesthetized at 6 h, 1 day, 3 days, and 7 days after I/R, and their brains were removed after perfusion with 4% paraformaldehyde. After gradient elution with sucrose, the brains were imbedded with OCT (Sakura Finetek, United States) and quickly frozen, and then coronally cut into 6 μm thick sections. The sections were permeabilized with 0.3% Triton X-100 for 15 min, retrieved with retrieval buffers for 10 min, and then blocked with 10% donkey serum for 1 h at 37°C. Next, the sections were incubated with Connexin43 antibody (1:200, Abcam, United States) or Aquaporin 4 Antibody (1:200, Abcam, United States) overnight at 4°C. The sections were then briefly washed with PBST and incubated with donkey anti-mouse secondary antibody (1:400, Abcam, United States) for 1 h at 37°C in the dark. After counterstaining with 4,6-diamidino-2-phenylindole (DAPI; Solarbio, Beijing, China), the sections were observed and photographed with fluorescent microscopy. Semi-quantitative analysis of the sections was conducted with the Image-J software.

### Real-Time Quantitative Reverse Transcription Polymerase Chain Reaction (RT-qPCR)

Total RNA was extracted by Trizol reagent (Invitrogen, United States). The RNA was then transcripted reversely into complementary deoxyribonucleic acid (cDNA) using a PrimeScript^TM^ RT reagent Kit (TAKARA, Japan), used as the template for polymerase chain reaction amplification. Quantitative RT-qPCR was conducted on a Light Cycler thermal cycler system (Bio-Rad, United States) using SYBR^®^ Premix Ex Taq^TM^ II (TAKARA, Japan). Gene-specific primers were used as follows: Cx43: The upstream primer: 5′−GGAAAGTACCAAACAGCAGCAG−3′, the downstream primer: 5′−CTGGGCACCTCTCTTTCACTT−3′, the amplified fragment was 152 bp; AQP4: The upstream primer: 5′−CATGGAGGTGGAGGACAACC−3′, the downstream primer: 5′−GCAGGAAATCTGAGGCCAGT−3′, the amplified fragment was 200 bp; GAPDH: The upstream primer: 5′−TGAAGAACAGGGAAGCAGCAA−3′, the downstream primer: 5′−ATCCAGTCCATTTTCCACCACA−3′, the amplified fragment was 200 bp. Amplification system: SYBR^®^ Premix Ex Taq^TM^ II 12.5 μl, Forward Primer (10 μm) 1 μl, Reverse Primer (10 μm) 1 μl, cDNA Template 2 μl, RNase FreedH_2_O 8.5 μl, and the final volume was 25 μl; amplification conditions: step 1: 95°C for 30 s, 1 cycle; step 2: 95°C for 5 s and 60°C for 30 s, 40 cycles; step 3: 95°C for 15 s, 60°C for 15 s, and 95°C for 15 s.

### Statistical Analysis

SPSS software (version 20.0) was used for data analyses; all data were expressed as mean ± standard deviation (SD). Differences between multiple groups were analyzed by One-way analysis of variance (ANOVA), while differences between two groups were analyzed by a *t*-test. Values of *P* < 0.05, are considered statistically significant.

## Results

### Neurological Deficits

**FIGURE 1:**
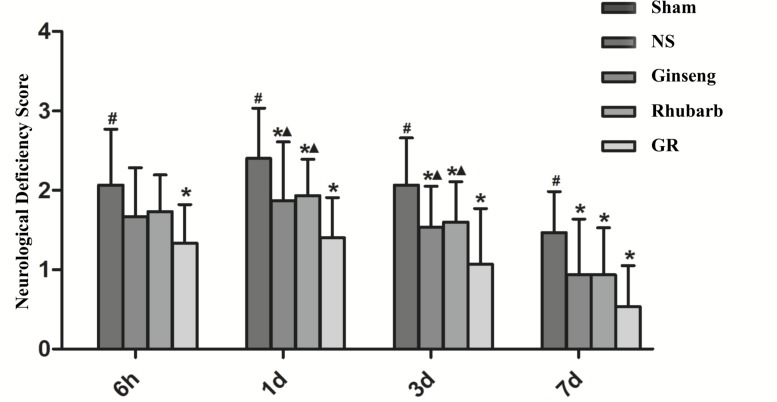
The Neurological deficiency score (NDS) in the sham group, normal saline (NS), Ginseng, Rhubarb, and the Ginseng + Rhubarb (GR) group at 6 h, 1 day, 3 days, and 7 days after I/R in rats (*n* = 5). ^#^*P* < 0.05, compared to the Sham group; ^∗^*P* < 0.05, compared to the NS group; ^▲^*P* < 0.05, compared to the GR group.

The sham group showed no neurological deficiency symptom, the NDS of the NS group was significantly increased from 6 h after I/R, and peaked at 1 day, then declined gradually. Compared with the NS group, the Ginseng and Rhubarb groups showed significantly lower NDS at 1 day, 3 days, and 7 days after I/R (*P* < 0.05). The GR group showed a significantly lower NDS at 6 h, 1 day, 3 days, and 7 days after I/R compared with the NS group (*P* < 0.05). Compared with the Ginseng and Rhubarb group, The GR group showed a significantly lower NDS at 1 day and 3 days after I/R (*P* < 0.05) (Figure 1).

### The Percentage of Cerebral Infarction Area

**FIGURE 2:**
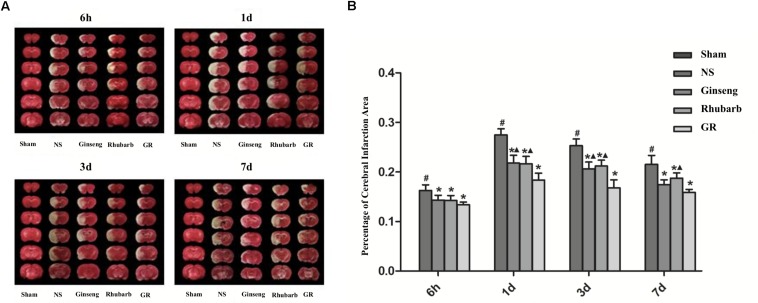
Infarction area assessments in the sham group, normal saline (NS), Ginseng, Rhubarb, and the Ginseng + Rhubarb (GR) group at 6 h, 1 day, 3 days, and 7 days after I/R in rats. **(A)** Triphenyltetrazolium chloride (TTC) staining. **(B)** Quantitative analysis for the percentage of the cerebral infarction area (*n* = 5). ^#^*P* < 0.05, compared with the Sham group; ^∗^*P* < 0.05, compared with the NS group; ^▲^*P* < 0.05, compared with the GR group.

The sham group showed no infarction area, the infarction area of the NS group was significantly increased from 6 h after I/R, and peaked at 1 day, then declined gradually. Compared with the NS group, all intervention groups showed significantly smaller infarction areas at 6 h, 1 day, 3 days, and 7 days after I/R (*P* < 0.05). Compared with the GR group, the Ginseng group showed a statistical difference at 1 days and 3 days after I/R, and the Rhubarb group showed a statistical difference at 1 day, 3 days and 7 days after I/R (*P* < 0.05) (Figures 2A,B).

### Expression of Cx43 Around Infarction Penumbra

**FIGURE 3:**
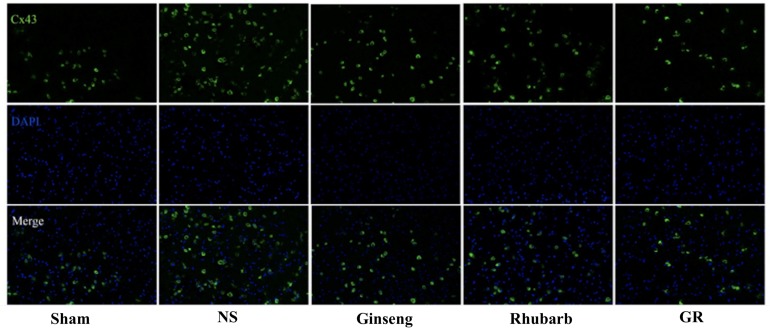
Immunofluorescence staining of the Cx43 protein around the infarction in the Sham, NS, Ginseng, Rhubarb, and the GR group at 1 day after I/R (*n* = 5).

**FIGURE 4:**
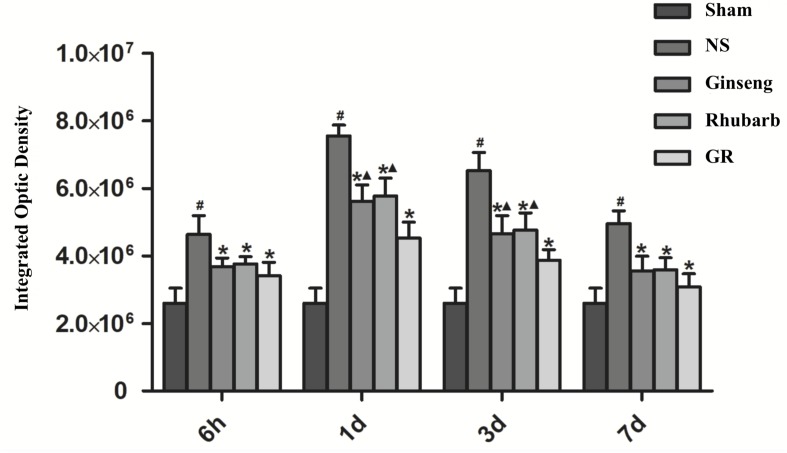
Quantitative analysis for the results of immunofluorescence staining of the Cx43 protein around the infarction in the Sham, NS, Ginseng, Rhubarb, and the GR group at 6 h, 1 day, 3 days, and 7 days after I/R (*n* = 5). ^#^*P* < 0.05, compared to the Sham group; ^∗^*P* < 0.05, compared to the NS group; ^▲^*P* < 0.05, compared to the GR group.

**FIGURE 5:**
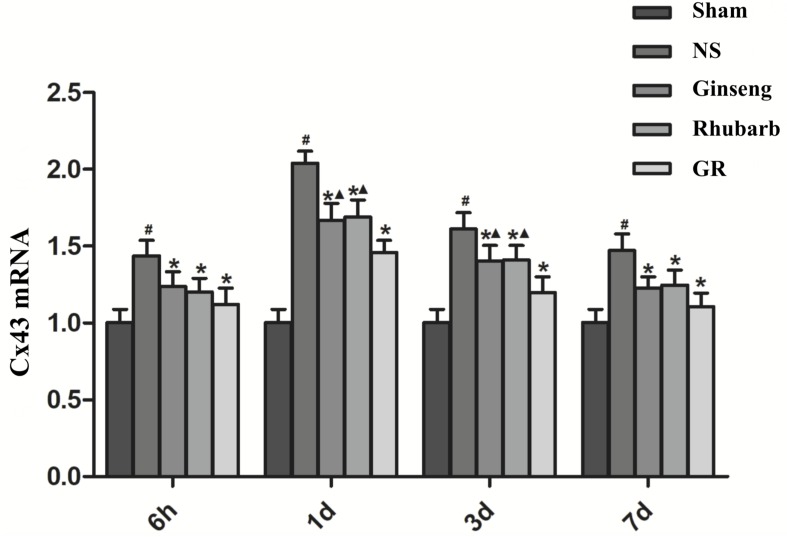
The Cx43 mRNA expression in the Sham, NS, Ginseng, Rhubarb, and the GR group at 6 h, 1 day, 3 days, and 7 days after I/R (*n* = 5). ^#^*P* < 0.05, compared to the Sham group; ^∗^*P* < 0.05, compared to the NS group; ^▲^*P* < 0.05, compared to the GR group.

Immunofluorescence and RT-q PCR showed that the expression of the Cx43 protein and mRNA was low in the sham group. The expression of the Cx43 protein and mRNA in the NS group increased at 6 h after I/R and peaked at 1 day, then declined gradually. Compared to the NS group, the intervention groups showed significantly lower expression of the Cx43 protein and mRNA at 6 h, 1 days, 3 days, and 7 days after I/R (*P* < 0.05). Compared with the Ginseng and Rhubarb group, the Cx43 protein and mRNA showed a statistically lower expression in the GR group at 1 day and 3 days after I/R (*P* < 0.05) (Figures 3–5).

### Expression of AQP4 Around Infarction Penumbra

**FIGURE 6:**
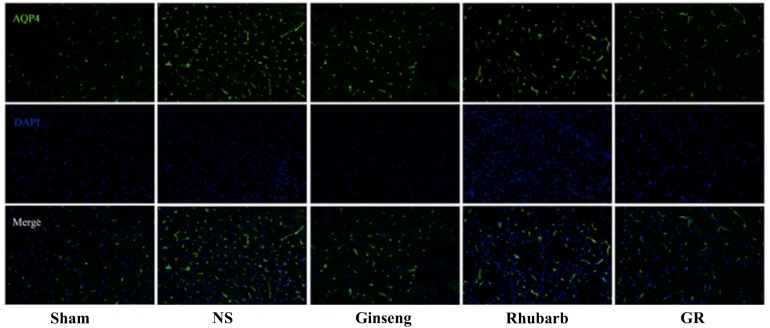
Immunofluorescence staining of the AQP4 protein around the infarction in the Sham, NS, Ginseng, Rhubarb, and the GR group at 1 day after I/R (*n* = 5).

**FIGURE 7:**
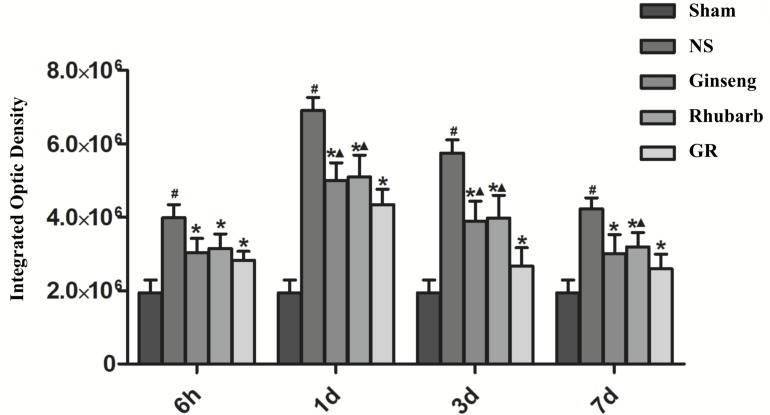
Quantitative analysis for the results of immunofluorescence staining of the AQP4 protein around the infarction in the Sham, NS, Ginseng, Rhubarb, and the GR group at 6 h, 1 day, 3 days, and 7 days after I/R (*n* = 5). ^#^*P* < 0.05, compared to the Sham group; ^∗^*P* < 0.05, compared to the NS group; ^▲^*P* < 0.05, compared to the GR group.

**FIGURE 8:**
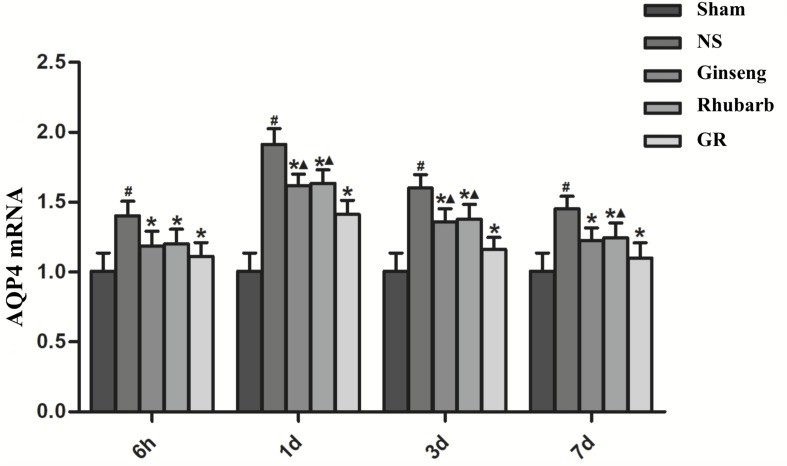
The AQP4 mRNA expression in the Sham, NS, Ginseng, Rhubarb, and the GR group at 6 h, 1 day, 3 days, and 7 days after I/R (*n* = 5). ^#^*P* < 0.05, compared to the Sham group; ^∗^*P* < 0.05, compared to the NS group; ^▲^*P* < 0.05, compared to the GR group.

Immunofluorescence and RT-q PCR showed that the expression of the AQP4 protein and mRNA was low in the sham group. The expression of the AQP4 protein and mRNA in the NS group increased at 6 h after I/R and peaked at 1 day, then declined gradually. Compared with the NS group, the intervention groups showed a significantly lower expression of the AQP4 protein and mRNA at 6 h, 1 day, 3 days, and 7 days after I/R (*P* < 0.05). Compared with the Ginseng group, the AQP4 protein and mRNA showed a statistically lower expression in the GR group at 1 day and 3 days after I/R (*P* < 0.05). Compared with the Rhubarb group, the AQP4 protein and mRNA showed a statistically lower expression in the GR group at 1 day, 3 days, and 7 days after I/R (*P* < 0.05) (Figures 6–8).

### Inhibitor CBX Reduce the NDS After I/R Injury

**FIGURE 9:**
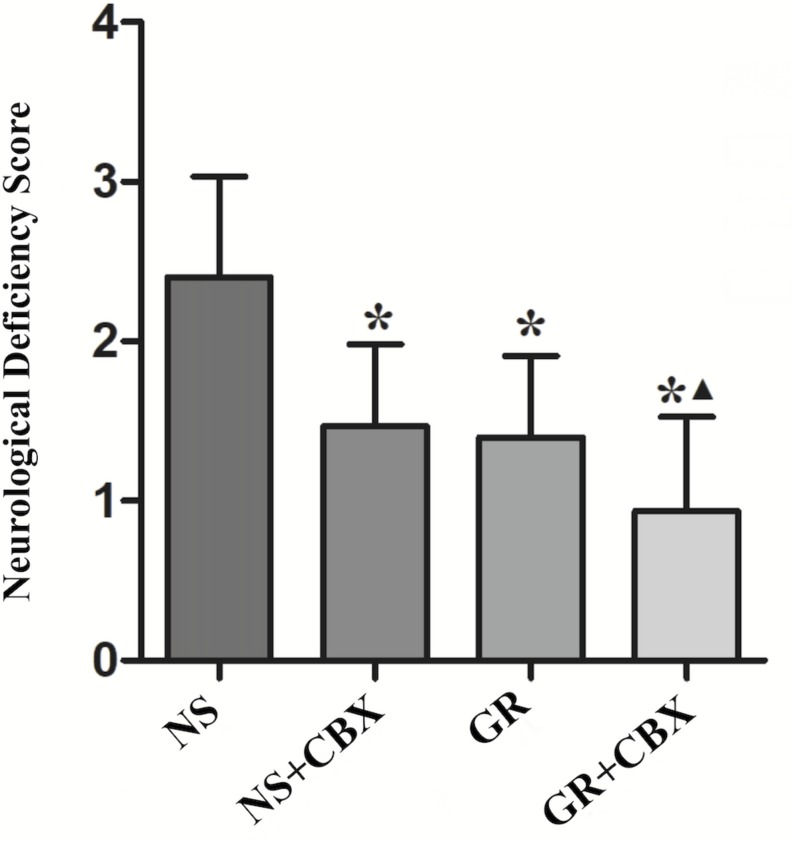
The NDS in the NS, Carbenoxolone (CBX), GR, and GR + CBX group at 1 day after I/R in rats (*n* = 5). ^∗^*P* < 0.05, compared to the NS group; ^▲^*P* < 0.05, compared to the GR group.

Compared to the NS group, the CBX group showed a significantly lower NDS 1 day after I/R (*P* < 0.05). Compared with the GR group, the GR + CBX group showed lower NDS 1 d after I/R (*P* < 0.05) (Figure 9).

### Inhibitor CBX Reduce the Infarction Area After I/R Injury

**FIGURE 10:**
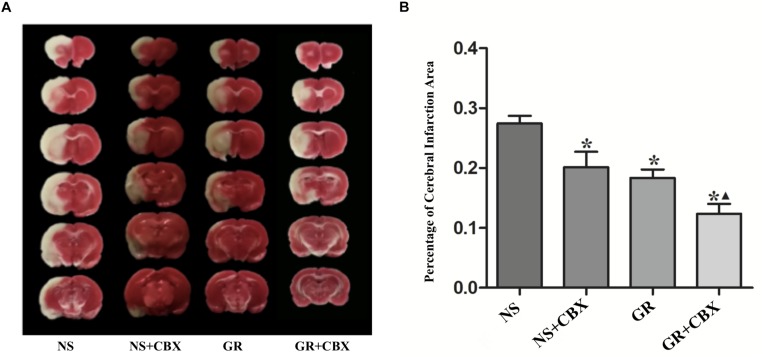
Infarction area assessments in the NS, CBX, GR, and GR + CBX group at 1 day after I/R in rats. **(A)** Triphenyltetrazolium chloride (TTC) staining. **(B)** Quantitative analysis for the percentage of the cerebral infarction area (*n* = 5). ^∗^*P* < 0.05, compared to the NS group; ^▲^*P* < 0.05, compared to the GR group.

Compared to the NS group, the CBX group showed a significantly smaller infarction area 1 day after I/R (*P* < 0.05). Compared to the GR group, the GR + CBX group showed a smaller infarction area 1 day after I/R (*P* < 0.05) (Figures 10A,B).

### Inhibitor CBX Down-Regulates the Expression of Cx43 and AQP4 After I/R Injury

**FIGURE 11:**
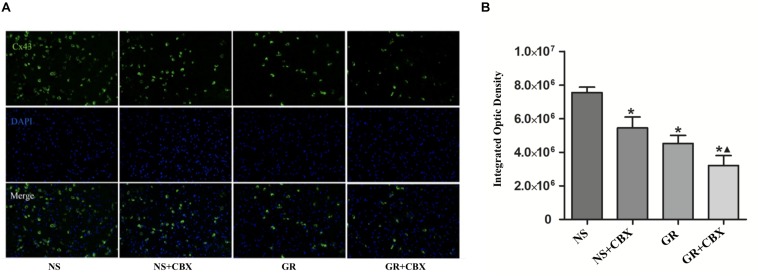
The expression of the Cx43 protein around the infarction in the NS, CBX, GR, and GR + CBX group at 1 day after I/R. **(A)** Immunofluorescence staining of the Cx43 protein. **(B)** Quantitative analysis for the results of immunofluorescence staining of the Cx43 protein (*n* = 5); ^∗^*P* < 0.05, compared to the NS group; ^▲^*P* < 0.05, compared to the GR group.

**FIGURE 12:**
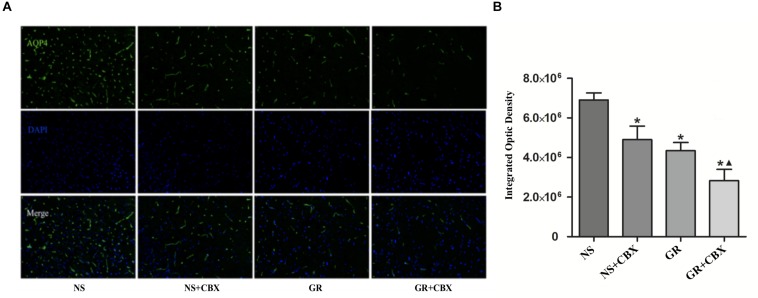
The expression of the AQP4 protein around the infarction in the NS, CBX, GR, and GR + CBX group at 1 day after I/R. **(A)** Immunofluorescence staining of the AQP4 protein. **(B)** Quantitative analysis of the results of immunofluorescence staining of the AQP4 protein (*n* = 5); ^∗^*P* < 0.05, compared to the NS group; ^▲^*P* < 0.05, compared to the GR group.

Immunofluorescence showed that compared to the NS group, the CBX group showed a significantly lower expression of the Cx43 and AQP4 protein 1 days after I/R (*P* < 0.05). Compared to the CBX group, the GR + CBX group showed a significantly lower expression of the Cx43 and AQP4 protein 1 day after I/R (*P* < 0.05). Compared to the GR group, the expression of the Cx43 and AQP4 protein was significantly lower in the GR + CBX group (*P* < 0.05) (Figures 11A,B, 12A,B).

**FIGURE 13:**
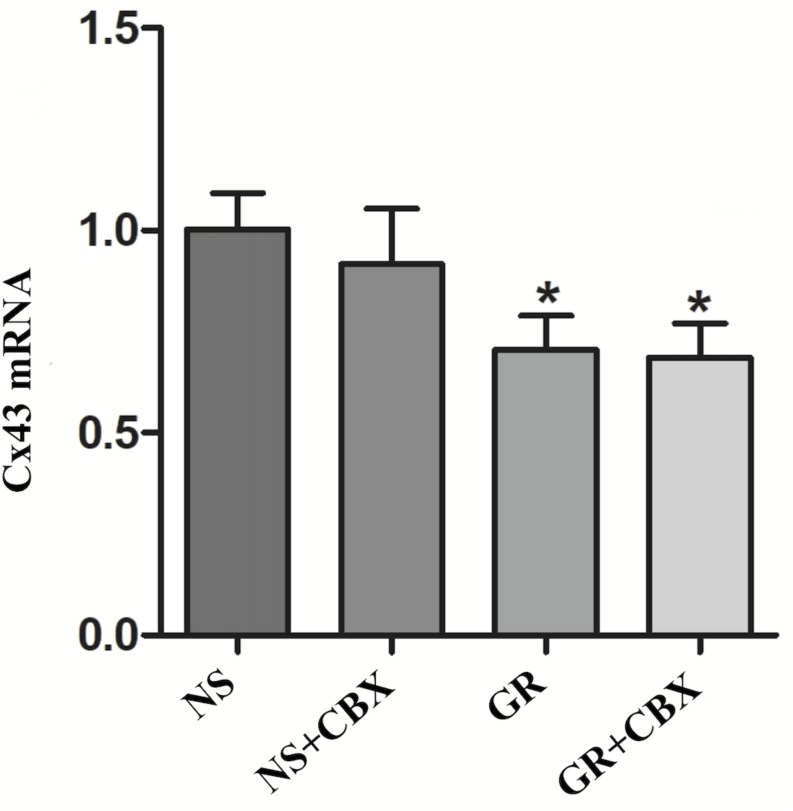
The Cx43 mRNA expression in the NS, CBX, GR, and GR + CBX at 1 day after I/R (*n* = 5). ^∗^*P* < 0.05, compared to the NS group; ^▲^*P* < 0.05, compared to the GR group.

**FIGURE 14:**
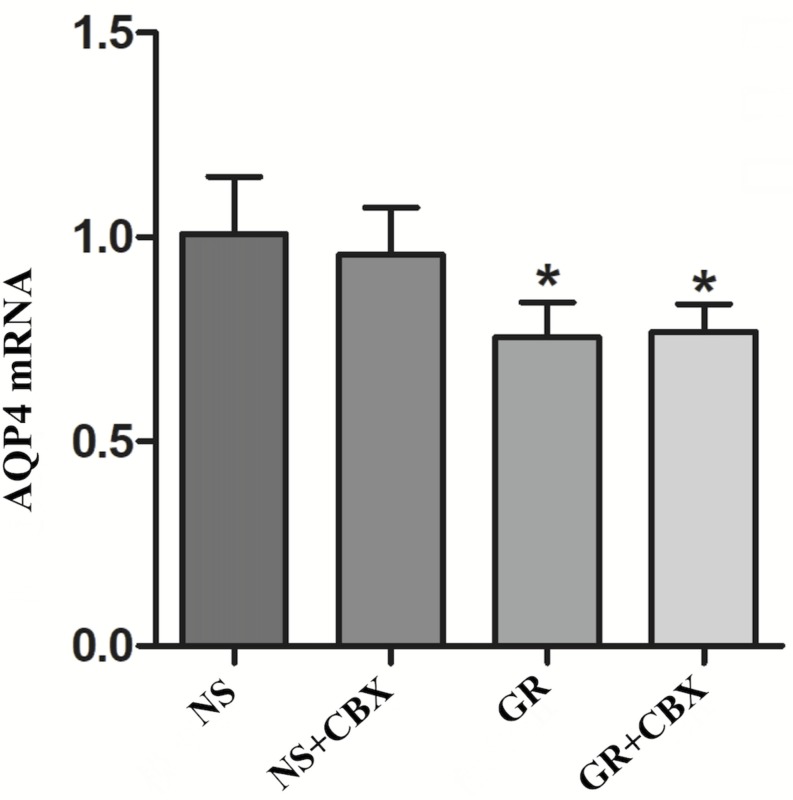
The AQP4 mRNA expression in the NS, CBX, GR, and GR + CBX at 1 day after I/R (*n* = 5). ^∗^*P* < 0.05, compared to the NS group; ^▲^*P* < 0.05, compared to the GR group.

RT-q PCR showed that compared to the NS group, the GR and GR + CBX group showed a significantly lower expression of the Cx43 and AQP4 mRNA 1 day after I/R (*P* < 0.05), while the CBX group showed no significant difference (*P* > 0.05); there was no significant difference in the expression of the Cx43 and AQP4 mRNA between the GR group and the GR + CBX group (*P* > 0.05) (Figures 13, 14).

## Discussion

Multi-herbal compatibility of formulas is the main function of TCM intervention, which uses two or more Chinese medicinal substances in combination, as per the pharmaceutical principle of TCM, according to patients’ pattern, herbal properties, and their interactions. The applications of herbal medicines through adequate compatibility can exert synergistic effects and reduce side effects and drug resistance (Zhou et al., 2017). Herbal pairings refer to the unique combination of two compatible herbs guided by the TCM principle, which is the most basic and the simplest form of multi-herbal formulas. It thus plays a fundamental and important role in the exploration of herbal compatibility (Wang et al., 2012). The present study showed the herbal compatibility of Ginseng and Rhubarb which has a synergistically neuroprotective effect in cerebral I/R rats, providing preclinical evidence of the herbal pairing in the treatment of ischemic stroke.

During cerebral ischemia and cerebral I/R injury, a series of pathological changes, such as oxidative stress injury, brain cell edema, inflammatory reaction and glutamate overload, will cause neuronal cell death and NVU damage (Jin et al., 2010; Chen et al., 2011), leading to further neurological impairment and increasing the infarction area (Mizuma and Yenari, 2017). After that, self-defense and self-repair will occur in the brain, attributing to angiogenesis, axon regeneration, and neural stem cell proliferation and differentiation. Neurological function will gradually improve, but will not be capable of completely returning to normal due to irreversible damage of the brain tissue. In this study, the five-point neurologic grading scale using Zea-Longa criteria (Longa et al., 1989) and the percentage of the infarction area were assessed in cerebral I/R rats, showing that the percentage of the infarction area and NDS increased at 6 h after I/R and peaked at 1 days, then decreased gradually but remained higher than that of the NS group at 7 days. The change trend of the cerebral infarction area percentage was basically consistent with the change of NDS, indicating that both the percentage of the cerebral infarction area and NDS could accurately reflect the severity and overall change trend of cerebral I/R injury. The present study indicates that the herbal compatibility of Ginseng and Rhubarb exerts synergistic effects on the functional recovery in rats with cerebral I/R injury.

A cerebral edema is a common pathological phenomenon in ischemic stroke, which may cause intracranial hypertension or even a cerebral hernia, leading to the death of patients (Walberer et al., 2008). AQP4 plays an important role in the formation and dissipation of a cerebral edema (Papadopoulos and Verkman, 2007), and has become a potential target for the treatment of cerebral edemas (Tang and Yang, 2016; Verkman et al., 2017). AQP4 is mainly distributed in brain parenchyma and between the fluid compartments (Papadopoulos and Verkman, 2013). During cerebral ischemic injury, AQPs regulate the permeability of the cell membrane mainly through the mitogen activated protein kinase pathway (Yang et al., 2013). When the permeability of blood vessels changes and the ion concentration gradient inside and outside the vessels and cell increases, AQP4’s own osmoreceptor can actively participate in water regulation after it is activated. Nito et al. (2012) found that the expression of AQP4 increased in cerebral I/R injured rats. Aoki et al. (2003) found that the expression of AQP4 mRNA was significantly increased around cerebral infarction. It can therefore be speculated that cerebral edema caused by cerebral ischemia may be related to the water transport involved in AQP4, which may be achieved by the upregulation of the AQP4 expression in astrocytes and the rapid transfer of water from the extracellular and mesenchymal to the intracellular. The knockout mouse model further verified that the deletion of the AQP4 gene can alleviate cerebral edema and blood–brain barrier (BBB) damage after cerebral ischemia, as well as reduce endothelial cell edema and cytotoxicity after saccharide and oxygen deprivation in brain tissue sections, indicating that AQP4 is involved in the formation of cytotoxic edema and BBB injury after cerebral ischemia (Katada et al., 2014). The present study indicates that monotherapy and combination therapy of Ginseng and Rhubarb have neuroprotective effects through the down-regulation of AQP4, while the combined use of Ginseng and Rhubarb could play a better protective role, which may be associated with the reduction of cerebral edema caused by ischemic stroke.

The function of GJ is related to the following aspects: (1) the expression of Cx protein; (2) the conformational change of Cx, opening or closing of the central aperture; (3) the changes of Cx distribution. During cerebral I/R injury, the transcription and expression of Cx43 in reactive astrocytes around the cerebral infarction can be increased (Haupt et al., 2007; Freitas-Andrade et al., 2017), and the conformation can be changed. Meanwhile, the gap connected half-channel can maintain an open state (Cotrina et al., 1998), and Cx43 can redistribute on the endfoot of astrocytes in the ischemic penumbra (Kondo et al., 1996). Some research found that the application of the GJ inhibitor can inhibit the function of Cx43 and alleviate cerebral I/R injury (Deng et al., 2014; Fan et al., 2015). The present study indicates that the change in Cx43 expression is consistent with the improvement of NDS and the decrease in the cerebral infarction area, suggesting that the monotherapy and combination therapy of Ginseng and Rhubarb have a neuroprotective effect via the down-regulation of Cx43; while the combined use of Ginseng and Rhubarb could play a better protective role.

Cx43 and AQP4 can interact in both structure and function. It has been demonstrated that Cx43 and AQP4 co-locate in the endfoot of astrocytes in the central nervous system and jointly participate in the transport of K+ from astrocytes to blood vessels, indicating that Cx43 and AQP4 are correlated at the level of water balance and GJ communication (Kong et al., 2008). AQP4 may be integrated with the astrocyte surface protein Cx43 and potassium channel Kir4.1 to eliminate excess fluid (Wu et al., 2013). Nicchia et al. (2005) found that astrocytes of AQP4 gene knockout mice significantly down-regulated Cx43, decreased cell coupling and cytoskeletal remodeling, suggesting that AQP4 and Cx43 on astrocytes may have a functional association. In addition, the absence of Cx43 also results in partial loss of AQP4 (Ezan et al., 2012; Li et al., 2015). Zhang et al. (2013) found that a intracerebroventricular injection of CBX can reduce the production of reactive oxygen species (ROS) and inhibit the activation of astrocytes and microglia, further reducing the cerebral infarction area in rats. It was found in an *in vitro* PC12 cell experiment that CBX can partially inhibit the opening of GJ and improve cell viability. The present experimental results found that CBX can inhibit GJ, reduce the expression of both the Cx43 and AQP4 protein, improve the NDS and reduce the percentage of the cerebral infarction area. However, this study mainly investigated the combined effect of Ginseng and Rhubarb on neuroprotection in I/R injury, without further research into the concentration-response relationship between AQP4 and Cx43, which is the limitation in this study as well as our future research directions.

## Conclusion

The herbal compatibility of Ginseng and Rhubarb has a synergistically neuroprotective effect during acute cerebral I/R injury, mainly by reducing the expression of Cx43 and AQP4.

## Data Availability

All datasets generated for this study are included in the manuscript/supplementary files.

## Author Contributions

W-TY and G-QZ designed the study. W-TY, YW, Y-HS, HF, ZX, and Q-QX performed the experiments and analyzed the data. W-TY and YW supervised the study and wrote the manuscript. All authors participated in the final approval of the version to be published.

## Conflict of Interest Statement

The authors declare that the research was conducted in the absence of any commercial or financial relationships that could be construed as a potential conflict of interest.
